# Supplementary material for: Pseudosparse neural coding in the visual system of primates
Source: Commun Biol. 2021 Jan 8;4:50. doi: 10.1038/s42003-020-01572-2 (PMC7794537; doi:10.1038/s42003-020-01572-2)
Supplement: Supplementary file 2 — Reporting Summary [file 42003_2020_1572_MOESM2_ESM.pdf]

natureresearch

Corresponding author(s): Sidney R. Lehky

Last updated by author(s): Dec 7, 2020

Reporting Summary

Nature Research wishes to improve the reproducibility of the work that we publish. This form provides structure for consistency and transparency in reporting. For further information on Nature Research policies, see Authors & Referees and the Editorial Policy Checklist .

Statistics

For all statistical analyses, confirm that the following items are present in the figure legend, table legend, main text, or Methods section.

n/a	Confirmed	
☐	☒	The exact sample size (*n*) for each experimental group/condition, given as a discrete number and unit of measurement
☒	☐	A statement on whether measurements were taken from distinct samples or whether the same sample was measured repeatedly
☐	☒	The statistical test(s) used AND whether they are one- or two-sided<br>*Only common tests should be described solely by name; describe more complex techniques in the Methods section.*
☒	☐	A description of all covariates tested
☒	☐	A description of any assumptions or corrections, such as tests of normality and adjustment for multiple comparisons
☐	☒	A full description of the statistical parameters including central tendency (e.g. means) or other basic estimates (e.g. regression coefficient) AND variation (e.g. standard deviation) or associated estimates of uncertainty (e.g. confidence intervals)
☐	☒	For null hypothesis testing, the test statistic (e.g. *F*, *t*, *r*) with confidence intervals, effect sizes, degrees of freedom and *P* value noted<br>*Give P values as exact values whenever suitable.*
☒	☐	For Bayesian analysis, information on the choice of priors and Markov chain Monte Carlo settings
☒	☐	For hierarchical and complex designs, identification of the appropriate level for tests and full reporting of outcomes
☐	☒	Estimates of effect sizes (e.g. Cohen's *d*, Pearson's *r*), indicating how they were calculated

Our web collection on statistics for biologists contains articles on many of the points above.

Software and code

Policy information about availability of computer code

Data collection	Custom C code
Data analysis	Matlab

For manuscripts utilizing custom algorithms or software that are central to the research but not yet described in published literature, software must be made available to editors/reviewers. We strongly encourage code deposition in a community repository (e.g. GitHub). See the Nature Research guidelines for submitting code & software for further information.

Data

Policy information about availability of data

All manuscripts must include a data availability statement. This statement should provide the following information, where applicable:

- Accession codes, unique identifiers, or web links for publicly available datasets
- A list of figures that have associated raw data
- A description of any restrictions on data availability

The data are available from the authors in response to reasonable requests.

Field-specific reporting

Please select the one below that is the best fit for your research. If you are not sure, read the appropriate sections before making your selection.

☒ Life sciences ☐ Behavioural & social sciences ☐ Ecological, evolutionary & environmental sciences

For a reference copy of the document with all sections, see nature.com/documents/nr-reporting-summary-flat.pdf

Life sciences study design

All studies must disclose on these points even when the disclosure is negative.

Sample size	Number of stimulus repetitions for each neuron and number of neurons collected was determined by the judgments of the various investigators in this reanalysis of several previously published data sets.
Data exclusions	Data from individual neurons were excluded if the number of stimulus repetitions did not reach a predetermined number. The reason insufficient data were collected from a neuron was typically because the recording microelectrode shifted away from the neuron due to small vibrations in the brain caused by heartbeat and breathing.
Replication	Data was collected from a total of eleven monkeys for different cortical areas. Each cortical data set contained data from one or two monkeys. Within each cortical data set stimulus response for each neuron was replicated an least five times, and more typically at least ten times.
Randomization	In each study, all monkeys in each cortical data set received all experimental conditions.
Blinding	Blinding was not relevant to our study as all monkeys in each cortical data set received all experimental conditions.

Reporting for specific materials, systems and methods

We require information from authors about some types of materials, experimental systems and methods used in many studies. Here, indicate whether each material, system or method listed is relevant to your study. If you are not sure if a list item applies to your research, read the appropriate section before selecting a response.

Materials & experimental systems

n/a	Involved in the study	
☒	☐	Antibodies
☒	☐	Eukaryotic cell lines
☒	☐	Palaeontology
☐	☒	Animals and other organisms
☒	☐	Human research participants
☒	☐	Clinical data

Methods

n/a	Involved in the study	
☒	☐	ChIP-seq
☒	☐	Flow cytometry
☒	☐	MRI-based neuroimaging

Animals and other organisms

Policy information about studies involving animals; ARRIVE guidelines recommended for reporting animal research

Laboratory animals	Macaque monkeys. M. mulatta, M. nemestrina, or M. fascicularis used in the various experiments included in the manuscript.
Wild animals	No wild animals were used.
Field-collected samples	There were no field-collected samples.
Ethics oversight	This manuscript involves a reanalysis of several previously published data sets collected in various institutions. The original published papers give details about the ethic committees involved.

Note that full information on the approval of the study protocol must also be provided in the manuscript.
